# Short-Term Outcomes of KeraSys Patch Graft for Glaucoma Drainage Devices: A Case Series

**DOI:** 10.1155/2013/784709

**Published:** 2013-03-17

**Authors:** Kundandeep S. Nagi, Ricardo J. Cumba, Nicholas P. Bell, Lauren S. Blieden, Alice Z. Chuang, Kimberly A. Mankiewicz, Donna Nguyen, Robert M. Feldman

**Affiliations:** ^1^Ruiz Department of Ophthalmology and Visual Science, The University of Texas Medical School at Houston, 6431 Fannin Street, MSB 7.024, Houston, TX 77030, USA; ^2^Department of Ophthalmology, The University of Texas Health Science Center at San Antonio, 7703 Floyd Curl Drive, Mail Code 6230, San Antonio, TX 78229, USA; ^3^Ophthalmology Department, Medical School, University of Puerto Rico, Medical Sciences Campus, P.O. Box 365067, San Juan, PR 00936, USA; ^4^Robert Cizik Eye Clinic, 6400 Fannin Street, Suite 1800, Houston, TX 77030, USA

## Abstract

*Purpose*. Tube-related exposure is a known complication of glaucoma drainage device (GDD) surgery. Our objective is to report the early (approximately 1 year) tube exposure rate of implants covered with a keraSys (IOP Inc., Costa Mesa, CA, USA) tissue reinforcement graft. *Patients and Methods*. A retrospective, noncomparative, consecutive case series of 42 eyes with GDD implantation with keraSys patch grafts was performed. Main outcome measurements included patch-related complications: patch exposure, tube exposure, wound dehiscence, and patch migration. *Results*. Forty-two eyes were followed for an average of 15.24 ± 10.44 months (range 1.0–32.3 months). Four (10%) eyes experienced patch-related complications: two with exposure 8 months postoperatively, one with exposure 13 months postoperatively, and one with exposure 4 weeks postoperatively. *Conclusion*. The effectiveness of the keraSys patch graft is limited by the higher than expected early exposure rate found in this case series. These results should be confirmed in other studies.

## 1. Introduction

Tube-related exposure is a known complication of glaucoma drainage device (GDD) surgery. The risk of infection [1, 2] and progressive exposure dictates prompt surgical intervention [3]. Different materials, including buccal mucosa [3], pericardium [4], human sclera [5], dura [6], and cornea [7], have been used to prevent exposure but none with universal success. In addition, there are several disadvantages particular to each material. Human sclera and pericardium are both opaque, which can be cosmetically unappealing to patients and interfere with certain early postoperative manipulations. Also, pericardium is thin, so some surgeons recommend folding it into double thickness to cover the tube. Buccal mucosa may result in pain at the harvest site postoperatively and requires increased surgical time. The use of dura and cornea carries an increased risk of infection, and cornea typically has to be thinned prior to use. 

KeraSys (IOP Inc., Costa Mesa, CA, USA) is bioengineered lamellar porcine small intestinal submucosa developed for the purpose of preventing GDD tube exposure while remaining cosmetically acceptable. KeraSys is clear, and the tube can be visualized through it. It is available commercially in sterile packaging with a long shelf life to allow for storage. Additionally, the cost of keraSys is typically significantly less (in the Houston market, approximately 50% less) than donor cornea, the other commonly used, and easily available, clear material. 

The use of keraSys has been previously reported in the repair of tube exposure, bleb revision, and for primary insertion in GDD surgery [8]. Cobb et al. have described the use of porcine small intestinal submucosa as a dura substitute in neurosurgery [9], and other groups have described its use in a number of other surgical fields [10–13]. However, there are no reports to our knowledge that describe the safety and efficacy of using keraSys to cover the subconjunctival portion of the GDD tube during initial surgery. Here, we report our short-term (approximately 1 year) outcomes of using keraSys as a tissue reinforcement graft for initial placement of GDDs.

## 2. Patients and Methods

A retrospective, noncomparative, consecutive case series of 42 eyes of 38 consecutive patients who underwent first time GDD surgery with keraSys patch grafts to cover the subconjunctival portion of the tube was performed. Relevant charts from the Robert Cizik Eye Clinic of the Ruiz Department of Ophthalmology and Visual Science at The University of Texas Medical School in Houston were identified by conducting a computerized search of the current procedural terminology code for initial implantation of glaucoma drainage device, 66180. All patients that underwent first time GDD surgery with a keraSys patch graft by 2 of the authors (RMF or NPB) between November 1, 2009 and November 30, 2010 were included. All cases during this time were performed using keraSys unless it was unavailable (see Section 3). Patients with previous GDD surgery were excluded from the study. The Institutional Review Board at The University of Texas Health Science Center at Houston (Committee for the Protection of Human Subjects) ruled this study to be exempt from review. This study adhered to the tenets of the Declaration of Helsinki and was HIPAA compliant.

### 2.1. Surgical Method

**Figure 1:**
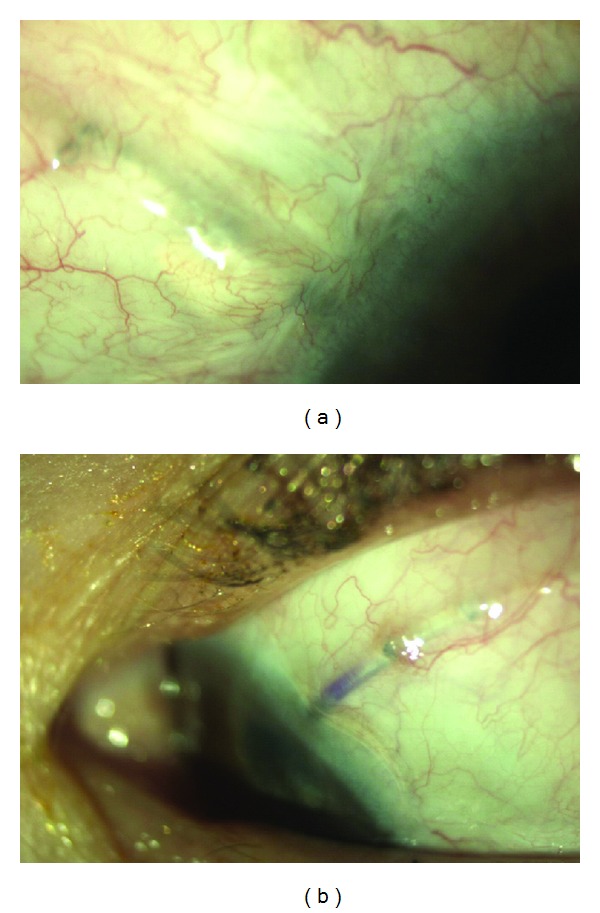
(a) Intact conjunctiva over graft. Note that the graft is not clearly visible. (b) Eroded tube in eye implanted with keraSys patch graft.

A keraSys patch graft was hydrated for 10 minutes in balanced salt solution as per manufacturer's instructions. It was then cut to the appropriate size and placed over the tube anteriorly to ensure coverage, where the tube enters sclera and at least a few millimeters posteriorly. An 8-0 polyglactin 910 (Vicryl, Ethicon Inc., Somerville, NJ, USA), 8-0 nylon, or 10-0 nylon suture was used to secure the patch to the sclera. Conjunctiva and Tenon's capsule were sewn into place to fully cover the keraSys graft (Figure 1). 

Patient demographics (age, gender, and race), preoperative clinical variables (hypertension, diabetes, or autoimmune disorders), intraocular pressure (IOP), type of glaucoma, previous ocular surgery or laser, pertinent ocular history, number of IOP-lowering medications, and surgical variables (date of procedure, type of device, combination procedure, placement of device, caliber and type of suture material used for the patch and conjunctiva) were collected. Postoperative data (vision, IOP, number of IOP-lowering medications, and patch-related complications) were collected at 1 week, 1 month, 3 months, 6 months, and 1 year of followup. Patch-related complications included patch exposure, tube erosion or exposure, wound dehiscence, and patch migration. 

Information was collected from chart review and entered into an Excel (Microsoft, Redmond, WA, USA) database with password protection. No patient identifiers were used. All data were kept confidential and only accessible to people involved in conducting this study. Data were summarized and compared using the paired *t*-test. All statistical analyses were performed using SAS for Windows 9.2 (SAS Institute Inc., Cary, NC, USA). A *P* value <0.05 was considered statistically significant. 

## 3. Results

Fifty-five eyes of 51 patients with initial implantation of a GDD were identified, with 13 eyes excluded from analysis due to previous GDD implantation or use of other patch graft material, leaving 42 eligible eyes from 38 patients. These 42 eyes were followed for an average of 15.24 ± 10.44 months, with a range of 1.0–32.3 months. Demographic information is described in Table 1. The mean age of all patients was 58.68 ± 16.69 years old (range 8–81). Overall, 39% (15 patients) were White, 34% (13 patients) Black, 11% (4 patients) Hispanic, 3% (1 patient) Asian, and 13% (5 patients) unknown. The most common ocular diagnosis was primary open angle glaucoma (POAG; 38%), followed by neovascular glaucoma (NVG; 31%), and inflammatory glaucoma (7%). Sixty-four percent of patients had previous intraocular surgery, with cataract surgery being the most common. Thirty-three eyes (79%) had Baerveldt implants (Abbott Medical Optics Inc., Santa Ana, CA, USA), while 9 eyes (21%) had Ahmed devices (New World Medical, Inc., Rancho Cucamonga, CA, USA). 

Preoperative best corrected visual acuity (BCVA) in 10 × logMAR scale (−log[BCVA] × 10) was 10.93 ± 8.73, with 15 eyes (36%) having count fingers or worse. The mean IOP prior to surgery was 33.37 ± 14.46 mm Hg on an average of 3.62 ± 0.85 IOP-lowering medications. At the 1 year followup visit (*N* = 27), 10 × logMAR vision did not show any statistically significant change (10.41 ± 10.27, *P* = 0.1942), though mean IOP decreased to 16.04 ± 6.85 mm Hg (statistically significant, *P* < 0.0001) on an average of 1.04 ± 1.32 medications (statistically significant, *P* = 0.0004). Followup vision, IOP, and medications data are presented in Table 2. 

The following 4 eyes (10%) experienced patch-related complications (Table 3).

- *ID no. 2*: 39-year-old female with uveitic glaucoma, 4 previous ocular surgeries, and a history of Retisert (Bausch & Lomb, Rochester, NY) implantation underwent simultaneous bilateral superotemporal GDD surgery with keraSys patch in both eyes. The patient was lost to followup for 9 months. Conjunctival thinning was noted in the left eye 12 months postoperatively. No conjunctival thinning or exposure was noted in the right eye. The patient was managed with lubrication and observation. On followup 1 month later, 13 months postoperatively, tube exposure was noted with a thinned (melting) keraSys graft. Surgical revision was promptly performed with a corneal patch graft without further complications.
- *ID no. 32*: 70-year-old diabetic, hypertensive, pseudophakic female with POAG had tube and nylon suture exposure through overlying conjunctiva that was discovered 8 months postoperatively, after being lost to followup for 6 months. Surgical revision was promptly performed without further complications.
- *ID no. 38*: 68-year-old diabetic, hypertensive female status post multiple intraocular surgeries with POAG underwent inferonasal GDD surgery with combined conjunctivoplasty (autologous conjunctival graft) due to lack of adequate conjunctiva to cover the tube. KeraSys patch exposure was noted in the early postoperative period (3 weeks) and was initially managed conservatively using lubrication and observation. The underlying tube was exposed 1 week later, and surgical intervention was required with no further complications.
- *ID no. 52*: 66-year-old female with NVG had tube exposure through the conjunctiva with no evidence of presence of the keraSys patch graft noted 8 months after her initial surgery. The patient was lost to followup for 7 months after her 1-month postoperative visit following the initial surgery. Prompt surgical revision with a corneal patch graft was performed with no further complications.

The average time to exposure for these 4 eyes was 202.5 ± 138.4 days, though 1 eye had a very early tube exposure at 29 days. All 4 eyes were from female patients, with an average age of 60.3 years. Two patients were White, one Black, and one Hispanic. Two of the 4 eyes that experienced patch-related complications were in diabetic, hypertensive patients who had POAG; the other patient was hypertensive but not diabetic and had NVG. Of the 3 eyes that had previous surgery, all 3 were pseudophakic, and one eye had previous trabeculectomy as well. Interestingly, a nylon suture was used to sew the keraSys patch to the sclera in 2 eyes. Polyglactin 910 was used in one eye, and suture type in the other was not noted in the operative report. See Table 3 for more information.

Two other eyes of the 42 total eyes (5%) had early postoperative conjunctival wound dehiscence. One had early (3 week) dehiscence of the conjunctival wound away from the graft and required no further surgical intervention. The other eye had early (1 week) dehiscence at the conjunctival incision near the graft requiring minor repair. No further exposures occurred in these eyes discussed above during the study period. No other eyes had exposure of patch material or tube, nor did they require any further surgical revision.

## 4. Discussion

Though a variety of materials have been purposed to prevent GDD exposure [3–6], none have been found to be universally reliable. Reports on efficacy are similarly variable, reflecting the lack of an ideal patch graft material. Lama and Fechtner reported 2 cases of early tube exposure with the use of a pericardial patch graft at 7 and 8 months [4], though Raviv et al. reported no tube exposures in a retrospective review of 44 eyes using the same patch material (mean followup was 10.2 ± 4.0 months) [14]. Smith et al. have described 3 cases of tube exposure in 2 eyes (3%, *N* = 62): 1 eye with dura at 6 months and 1 eye with sclera at 15 months and in the same eye with pericardium 21 months later (36 months). However, the authors were unable to find a clear indication to choose a particular patch material over the others with respect to tube exposure [15]. A recent study by Anand et al. describes the use of 300 micron thick amniotic membrane as an initial patch graft and reports 1 eye (2%) with tube exposure out of a total of 42 eyes [3]. 

In several large studies, the rate of tube exposure ranges from 0 to 3% at 1 year of followup. In the Tube Versus Trabeculectomy (TVT) Study, no instances of tube exposure in 107 cases were reported at 1 year of followup [16]. In the Ahmed Baerveldt Comparison (ABC) Study, 3 tube exposures (1.1%) were reported at 1 year of followup in 276 cases [17]. In the Ahmed Versus Baerveldt (AVB) Study, 5 cases of tube exposure (2%) in 238 cases were reported at 1 year [18]. A meta-analysis of prior GDD studies by Stewart et al. reported an overall incidence of 2.0 ± 2.6% (total of 3255 eyes), an average exposure per month rate of 0.09 ± 0.14% [19]. 

In our case series, 4 of 42 eyes (10%) experienced exposure with externalization of the tube through the overlying keraSys patch within approximately 1 year. The exposure occurred with an average of 202.5 days after the initial tube shunt procedure, though one complicated eye had very early exposure within 1 month of surgery. Even with the inclusion of the very early exposure, the mean time to exposure is likely overestimated given that 2 of the 4 patients were lost to followup, probably only returning due to discomfort related to exposure of uncertain duration. 

There are several interesting observations in those eyes that experienced tube exposure. Two of the eyes were in diabetic, hypertensive patients with previous intraocular surgery. One eye had no previous intraocular surgery, but the patient was hypertensive and diagnosed with neovascular glaucoma. The final patient was diagnosed with uveitic glaucoma, had 4 previous ocular surgeries, and was 37 years of age. She was not on systemic immunosuppressives. Nylon suture material was used to secure the patch to the sclera in 2 of the 4 eyes. 

There are several limitations to this study. First, given its design and relatively small sample size, no direct comparison can be made to determine the safety and efficacy of keraSys in comparison to other materials. Also, because the GDD implantation procedure was not standardized, slight differences in technique were not accounted for. There are also limitations inherent to a retrospective study, including incompletely documented data in the charts reviewed. Because the study is not intended to or designed to compare keraSys directly with other treatments, any conclusions from this study that purport that this treatment modality is superior or inferior to any other treatments cannot be made. While longer followup should be evaluated with keraSys, to our knowledge, this paper is the largest case series describing the patch-related complications of this novel material when used in primary GDD implantation. 

We report our early (approximately 1 year) experience with keraSys, bioengineered lamellar porcine small intestinal mucosa, as a material to cover the subconjunctival portion of GDD tubes. The effectiveness of the keraSys patch graft in our case series is limited due to the high exposure rate found. Further study is required to determine whether continued use of keraSys for this purpose is warranted. 

## Figures and Tables

**Table 1:** Demographics, medical history, and baseline characteristics.

Variable	Summary Statistics
Demographics	

Age, years; mean (SD)	58.68 (16.69)
Sex, no. of female (%)	25 (66)
Race/ethnicity, no. of participants (%)	
Black	13 (34)
White	15 (39)
Hispanic	4 (11)
Asian	1 (3)
Unknown	5 (13)

Medical and ocular history	

Hypertension, no. of participants (%)	25 (66)
Diabetes mellitus, no. of participants (%)	14 (37)
Autoimmune diseases, no. of participants (%)	2 (5)
Preoperative visual acuity	
Visual acuity, 10 × logMAR; mean (SD)	10.93 (8.73)
Visual acuity, 20/40 or better; no. of eyes (%)	12 (29)
Visual acuity, count figures or worse; no. of eyes (%)	15 (36)
Diagnosis, no. of eyes (%)	
Primary open angle glaucoma (POAG)	16 (38)
Neovascular glaucoma (NVG)	13 (31)
Inflammatory glaucoma	3 (7)
Other	10 (24)
Preoperative IOP, mm Hg; mean (SD)	33.37 (14.46)
Preoperative IOP-lowering medications, no.; mean (SD)	3.62 (0.85)
Previous ocular surgery, no. of eyes (%)	27 (64)
Glaucoma surgery	13 (31)
Trabeculectomy	11 (26)
Panretinal photocoagulation	11 (26)
Cyclophotocoagulation	1 (2)
Selective laser trabeculoplasty	5 (12)
Retina surgery	5 (12)
Vitrectomy	2 (5)
Cataract surgery	20 (48)
Posterior chamber intraocular lens	18 (43)
Anterior chamber intraocular lens	2 (5)

Tube shunt implantation parameters	

Type of glaucoma drainage device implanted, no. of eyes (%)	
Baerveldt 350	30 (71)
Baerveldt 250	3 (7)
Ahmed FP7	8 (19)
Ahmed S2	1 (2)
Shunt placement, no. of eyes (%)	
Inferior nasal	2 (5)
Superior temporal	40 (95)
Conjunctival suture, no. of eyes (%)	
8-0 nylon	1 (2)
8-0 polyglactin 910	39 (93)
Unknown	2 (5)
Patch suture, no. of eyes (%)	
8-0 nylon	4 (10)
8-0 polyglactin 910	23 (55)
10-0 nylon	10 (24)
10-0 polyglactin 910	1 (2)
Unknown	4 (9)
Plate anchor suture, no. of eyes (%)	
5-0 nylon	2 (5)
8-0 nylon	33 (79)
9-0 nylon	2 (5)
10-0 nylon	1 (2)
5-0 polyethylene terephthalate	4 (10)
Tube occlusion suture, no. of eyes (%)	
8-0 nylon	33 (79)
Unknown	9 (21)
Obturator suture, no. of eyes (%)	
3-0 polypropylene	31 (74)
Unknown	11 (26)
Tube anchor suture, no. of eyes (%)	
8-0 nylon	36 (86)
10-0 nylon	4 (10)
Unknown	2 (5)

IOP: intraocular pressure; SD: standard deviation.

**Table 2:** Followup vision, IOP, and number of medications.

Followup	No. of eyes examined	Vision (10 × logMAR)	IOP (mm Hg)	No. of IOP-lowering medications
Baseline	42	10.93 ± 8.73	33.37 ± 14.46	3.62 ± 0.85
1 week	40	11.03 ± 8.80	22.68 ± 11.89*	1.08 ± 1.44*
1 month	42	9.79 ± 8.15	24.81 ± 10.07*	1.57 ± 1.56*
3 months	39	8.49 ± 8.62*	17.92 ± 5.72*	0.74 ± 1.12*
6 months	29	9.90 ± 9.81	14.97 ± 5.14*	1.14 ± 1.19*
12 months	27	10.41 ± 10.27	16.04 ± 6.85*	1.04 ± 1.32*

*Significantly different from baseline.

IOP: intraocular pressure.

**Table 3:** Characteristics of eyes with tube exposure.

Characteristics	ID = 2	ID = 32	ID = 38	ID = 52
Gender	F	F	F	F
Race	W	H	B	W
Diagnosis	UG	POAG	POAG	NVG
Hypertension	N	Y	Y	Y
Diabetes mellitus	N	Y	Y	N
Systemic autoimmune disease	N	N	N	N
Age (yrs)	37	70	68	66
Preoperative BCVA	20/400	20/40	20/50	Count Fingers
Preoperative IOP (mm Hg)	42	18	41	30
No. of previous ocular surgeries	4	1	3	0
No. of previous trabeculectomies	0	0	1	0
Cataract surgery	PCIOL	PCIOL	PCIOL	N
Type of GDD	B-250	B-350	B-350	B-350
Patch suture	8-0V	10-0N	Unknown	10-0N
Days until exposure	390	260	29	247

F: female; H: Hispanic; B: Black; W: White; UG: uveitic glaucoma; POAG: primary open angle glaucoma; NVG: neovascular glaucoma; N: no; Y: yes; BCVA: best corrected visual acuity; PCIOL: posterior chamber intraocular lens; 8-0V: 8-0 polyglactin 910; 10-0N: 10-0 nylon; GDD: glaucoma drainage device; B-350: Baerveldt glaucoma implant with 350 mm^2^ plate; B-250: Baerveldt glaucoma implant with 250 mm^2^ plate; IOP: intraocular pressure.
